# Vav1 Sustains the In Vitro Differentiation of Normal and Tumor Precursors to Insulin Producing Cells Induced by all-Trans Retinoic Acid (ATRA)

**DOI:** 10.1007/s12015-020-10074-x

**Published:** 2020-11-09

**Authors:** Federica Brugnoli, Silvia Grassilli, Vincenzo Cardinale, Guido Carpino, Eugenio Gaudio, Domenico Alvaro, Silvano Capitani, Valeria Bertagnolo

**Affiliations:** 1 Section of Anatomy and Histology, Department of Morphology, Surgery and Experimental Medicine, University of Ferrara, Via Fossato di Mortara, 70, 44121 Ferrara, Italy; 2 LTTA Centre, University of Ferrara, Ferrara, Italy; 3 Department of Medico-Surgical Sciences and Biotechnologies, Sapienza University of Rome, Rome, Italy; 4 Department of Movement, Human and Health Sciences, Division of Health Sciences, University of Rome “Foro Italico”, Rome, Italy; 5 Division of Human Anatomy, Department of Anatomical, Histological, Forensic Medicine and Orthopedics Sciences, Sapienza University of Rome, Rome, Italy; 6 Department of Medicine and Medical Specialties, Sapienza University of Rome, Rome, Italy

**Keywords:** Vav1, Insulin producing cells, Human biliary tree stem/progenitor cells (hBTSCs), Pancreatic ductal adenocarcinoma (PDAC) cells, All-*trans* retinoic acid (ATRA)

## Abstract

All-*trans* retinoic acid (ATRA) promotes the development and the function of insulin producing cells and induces partial differentiation of pancreatic tumor cells. A number of evidences clearly indicate that the ATRA mediated signaling may have a substantial role in therapeutic approaches based on restoration of functional β-cells. Among the proteins up-regulated by ATRA, Vav1 is involved in maturation and function of haematopoietic cells and is essential for retinoids induced differentiation of tumor promyelocytes. The presence of Vav1 in solid tissues, including pancreas, is considered ectopic and no role in the differentiation of human epithelial cells has so far been described. We demonstrated here that Vav1 sustains the maturation to β-cells of the normal precursors human Biliary Tree Stem/progenitor Cells (hBTSCs) induced by a differentiation medium containing ATRA and that, in the mature normal pancreas, insulin-producing cells express variable levels of Vav1. Using pancreatic ductal adenocarcinoma (PDAC)-derived cells, we also revealed that the ATRA induced up-modulation of Vav1 is essential for the retinoid-induced trans-differentiation of neoplastic cells into insulin producing cells. The results of this study identify Vav1 as crucial molecule in ATRA induced maturation of insulin producing cells and suggest this protein as a marker for new strategies ended to restore functional β-cells.

**Figure:**
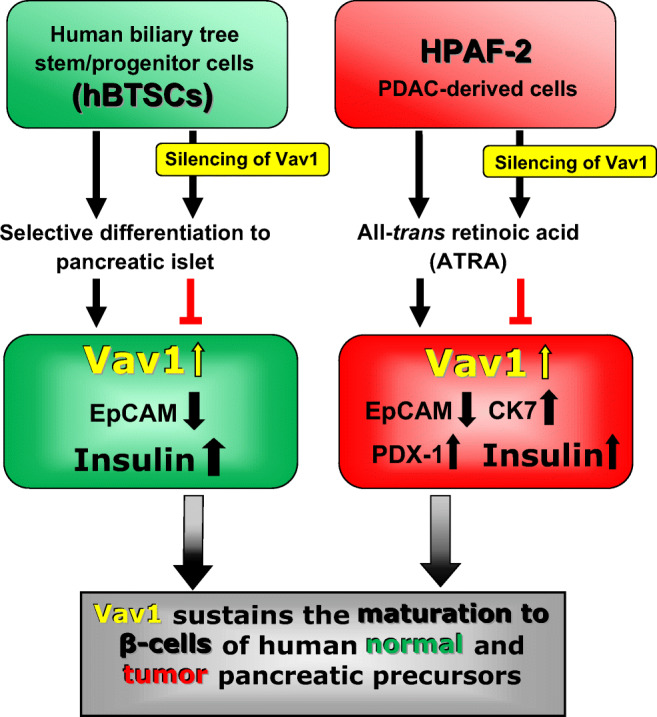
Graphical abstract

Graphical abstract

## Introduction

All-*trans* retinoic acid (ATRA) is a physiologically active member of the metabolites of vitamin A that mediates multiple signaling pathways associated with cell homeostasis, proliferation, and differentiation [1, 2]. During embryogenesis, retinoids are required for organogenesis of central nervous system, lung, kidney, intestine, and pancreas [2, 3]. Although conflicting data exist on the distinct role of retinoids in endocrine and exocrine differentiation of pancreas, ATRA favors the development of insulin positive cells in endocrine clusters [4, 5] and maintains pancreatic endocrine functions, suggesting that it can be used to improve diabetes management [6].

After the pioneering evidence that ATRA is a successful option in the treatment of acute promyelocytic leukemia (APL) [7, 8], over the last decades large interest has been attracted in studying the potential use of this retinoid in solid tumors, including pancreatic cancer [9, 10]. In cells from pancreatic ductal adenocarcinoma (PDAC), ATRA induces cell-cycle arrest, apoptosis, and epithelial differentiation [11, 12] and drives in vitro trans-differentiation into functional insulins secreting cells [13].

ATRA mediates its function through specific receptors, which belong to the ligand-dependent transcription factor superfamily of nuclear hormone receptors and are involved in the transcriptional regulation of over 530 different genes [14]. Among them, the gene codifying for Vav1, a multidomain protein physiologically expressed only in haematopoietic cells where it is essential for both maturation and function [15, 16]. Vav1 is up-regulated by ATRA in cells from APL and plays a crucial role in the differentiation of tumoral promyelocytes induced by retinoids [17–19].

Aberrant expression of Vav1 in non-hematopoietic cells was almost always associated with the appearance of a tumor phenotype [20–25] and, with the exception of breast tumors [26], with a negative tumor prognosis [25, 27]. Even if its prognostic role is controversial, the expression of Vav1 in pancreatic cancer was correlated to signal transduction processes responsible for aggressive malignant phenotypes [21, 28, 29].

As one of the most important roles of retinoic acid in pancreas is the regulation of differentiation both in normal and pathological states, aim of this work was to establish if Vav1 is one of the molecules through which retinoids act at pancreatic level. Our results revealed an unprecedented role of Vav1 in sustaining the ATRA mediated differentiation of normal and tumor cells to insulin producing cells.

## Materials and Methods

### Cell Culture and Treatments

All reagents were from Sigma (St Louis, MO) unless otherwise indicated.

Human biliary tree stem/progenitor cells (hBTSCs) were isolated and cultured in Kubota’s medium (KM) at 37 °C in a humidified atmosphere of 5% CO_2_ in air as previously described [30]. Three different modified KM were used to induce selective differentiation of hBTSCs to Hepatocytes (HM), Cholangiocytes (CM) and Pancreatic islet (PM, containing 1 μM ATRA), as previously described [30].

The pancreatic cancer cell lines HPAF-2, HPAC and PL-45 were purchased from the American Type Culture Collection (Rockville, MD) and were cultured in Dulbecco’s modified Eagle’s medium (DMEM, Gibco Laboratories, Grand Island, NY) supplemented with 10% fetal bovine serum (FBS, Gibco Laboratories), 1% L-Glutamine and 1% penicillin-streptomycin solution (Gibco Laboratories) and grown at 37 °C in a humidified atmosphere of 5% CO_2_ in air.

HPAF-2, HPAC and PL-45 cells were cultured in the presence of ATRA or vehicle (ethanol) for 96 h. Cells were daily counted by means of a hemocytometer in the presence of trypan blue, in order to determine the number of viable cells.

The morphology of hBTSCs and HPAF-2 cells under the different experimental conditions was analyzed with an inverted phase-contrast microscope (Eclipse TE2000-E, Nikon S.p.a., Melville, NY) acquiring cell images by the ACT-1 software for a DXM1200F digital camera (Nikon).

### Modulation of Vav1 Expression

To down-modulate Vav1 expression in hBTSCs differentiating to pancreatic islet, precursors cells seeded in chamber slides were subjected to transient transfection with 1 mg/ml Lipofectamine 2000 (Thermo Fisher Scientific Inc., Waltham, MA) and 100 pmol of Vav1 siRNAs (Santa Cruz Biotechnology). Transfection procedure was repeated every 4 days of culture in PM.

For modulation of Vav1 expression in HPAF-2, exponentially growing cells were transfected with a mixture of Vav1 siRNAs (Santa Cruz Biotechnology, Santa Cruz, CA) or with a pEF plasmid expressing the human full-length Vav1, following previously reported procedures [31].

### Immunochemical and Immunocytochemical Analysis

Total lysates from hBTSCs and cell lines under different experimental conditions were separated on 8.5% polyacrylamide denaturing gels and blotted to nitrocellulose membranes (GE Healthcare Life Science, Little Chalfont, UK) that were reacted with antibodies directed against Vav1 and/or PDX-1 (Santa Cruz Biotechnology) and against β-Tubulin, following the manufactures instructions. The immunocomplexes were detected using the ECL system (Perkin-Elmer, Boston, MA) and the chemiluminescence bands were acquired with ImageQuant TM LAS 4000 biomolecular imager (GE Healthcare Life Science) and quantified by densitometrical analysis (ImageQuant TL software, GE Healthcare Life Science).

For immunocytochemical analysis of Insulin in hBTSCs, cells grown on chamber slides were fixed with freshly prepared 4% paraformaldehyde, washed once in PBS, and incubated with the specific primary antibody for 3 h in NET gel [32] at room temperature. After 3 washes, samples were reacted with a FITC-conjugated secondary antibody and fluorescent samples were analyzed with a Nikon Eclipse TE2000-E microscope (Nikon).

Immunocytochemical analysis of Vav1, Cytokeratin 7 and Insulin (Santa Cruz Biotechnology) and of EpCAM (NCL-ESA, Leica, I) in HPAF-2 cells was performed essentially following a previously described procedure [26].

Images of fluorescent cells were acquired with the ACT-1 software for a DXM1200F digital camera (Nikon) and the staining intensity was measured with the ImageJ software (http://rsb.info.nih.gov/ij/).

### Cytofluorimetrical Evaluation of EpCAM Expression

The expression of EpCAM in hBTSC was evaluated by flow cytometry by direct staining with a fluorescein isothiocyanate (FITC)-conjugated anti-CD326 (EpCAM) monoclonal antibody (Miltenyi Biotec, Bologna, I), as suggested by manufacturer’s instructions. To assess the role of Vav1 on EpCAM levels, this surface antigen was evaluated in hBTSCs subjected to silencing of Vav1 with specific siRNAs during the first or the last 7 days of pancreatic differentiation. Non-specific fluorescence was assessed by using an isotype-matched control, Mouse IgG1-PE (Immunotech, Coulter Company, Marseille, F) and 7-amino-actinomycin D (Becton Dickinson, San Josè, CA) was added to the samples to exclude dead cells. All the samples were then analyzed by a FACSCalibur flow cytometer (Becton Dickinson) with the CellQuest Pro 6.0 software (Becton Dickinson). Data collected from 10,000 cells are shown as a percentage of positive cells.

### Immunohistochemical Analysis

Immunohistochemical analysis of Vav1 in formalin-fixed paraffin embedded samples of human normal pancreatic tissue (kindly provided by Dr. Liberatore, SS Annunziata Hospital, Chieti, I) was performed as previously reported [26].

For immunofluorescence analysis of Vav1 and insulin, tissue samples were subjected to antigen retrieval in citrate buffer (10 mM, pH 6.0) and permeabilized in TBS buffer containing 4% BSA and 0.3% Triton. Samples were then reacted with a mixture of primary antibodies directed against Vav1 and insulin, ON at 4 °C in humidified chamber. After 3 washes with TBS, samples were incubated for 1 h at room temperature with a mixture of FITC and TRITC-conjugated secondary antibodies diluted in TBS containing 4% BSA and 0.3% Triton. Slides were counterstained with DAPI, dehydrated through graded alcohols and mounted with the anti-fade mounting medium Glycerol DABCO. Negative controls were obtained by omitting the primary antibodies.

Each tissue sample was analyzed by a Nikon Ci-L microscope (Nikon) and cell images were acquired with the NIS-ELEMENTS D software for a DS-Qi2Mc digital camera (Nikon).

### Statistical Analysis

The results were expressed as means ± standard deviations of three independent experiments. Statistical analysis was performed by using the two-tailed Student’s t test for unpaired data with the GraphPad Prism 6.0 statistical package (GraphPad Software, San Diego, CA) and *P* values <0.05 were considered statistically significant.

## Results

### Vav1 Is Necessary for Differentiation of Pancreatic Progenitor Cells to β-Cells

**Fig. 1:**
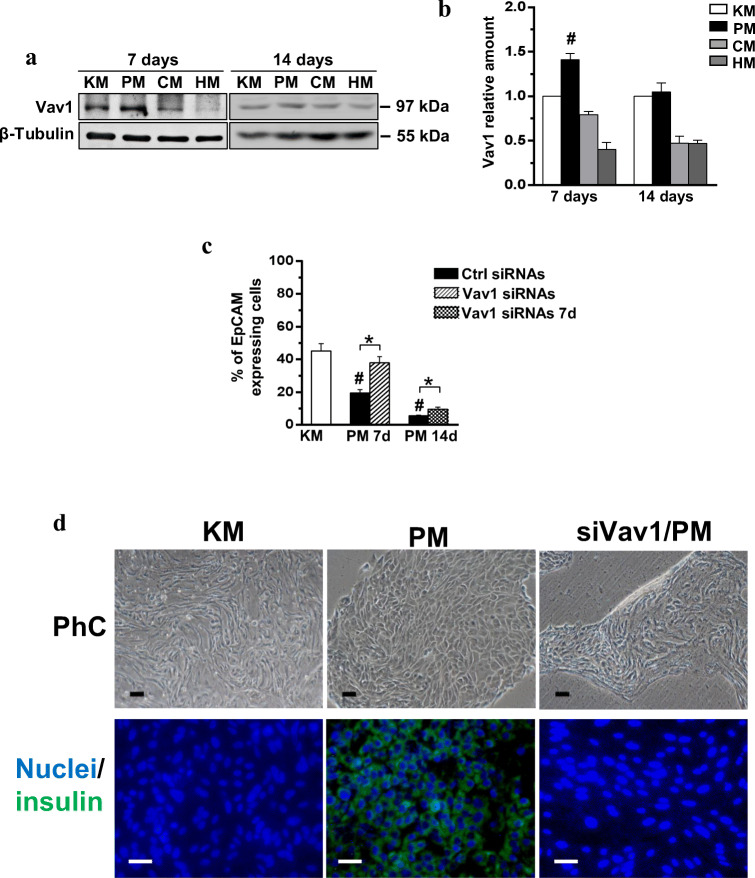
**Vav1 sustains differentiation of hBTSCs toward pancreatic β-cells.** (**A**) Representative Western blot analysis with the reported antibodies of lysates from hBTSCs grown in Kubota’s medium (KM) or in the presence of a medium able to induce selective differentiation to pancreatic islet (PM), cholangiocytes (CM) and hepatocytes (HM) for the indicated times (days). (**B**) Levels of Vav1 as deduced from the densitometry of immunochemical bands normalized with β-Tubulin, used as internal control for equivalence of loaded proteins. (**C**) Cytofluorimetrical evaluation of EpCAM surface expression after labelling with a FITC-conjugated specific antibody in hBTSCs growing in KM, cultured in PM, silenced for Vav1 and cultured in PM for 7 days (Vav1 siRNAs) and cultured for 7 days in PM and subsequently grown in PM for additional 7 days in the presence of Vav1 siRNAs (Vav1 siRNAs 7d). Cells positive for EpCAM expression are reported as percentage. All the data are the mean of three separate experiments performed in triplicate ±SD. **#** *P* < 0.05 versus control; * *P* < 0.05 between bars. (**D**) Representative images of hBTSCs growing for 14 days on chamber slides in the presence of KM, cultured in PM and silenced for Vav1 during PM differentiation (siVav1/PM). PhC: phase contrast images; Nuclei/Insulin: merged immunocytochemical images of insulin (green) and nuclear (blue) staining. Bar = 50 μm

The possible role of Vav1 in modulating the differentiation of normal precursors to β-cells was investigated in hBTSCs induced to differentiate toward functional β-cells. As shown in Fig. 1A, Vav1 is expressed in hBTSCs and slightly increased after 7 days of culture in pancreas differentiation medium (PM). After 14 days of differentiation culture, sufficient to induce functional β-cells [30], the levels of Vav1 were similar to those found in hBTSCs grown in control medium (Fig. 1A, B). HBTSCs cells grown in a medium able to induce their differentiation to hepatocytes and colangiocytes revealed a time-dependent decrease of Vav1 expression (Fig. 1A, B).

To assess the role of Vav1 in maturation of hBTSCs to β-cells, its expression was silenced by specific siRNAs during pancreatic differentiation. The cytofluorimetrical analysis of EpCAM positive cells showed, as expected [30], that differentiation is accompanied by a time dependent loss of this stem cells marker (Fig. 1C). The silencing of Vav1 expression during the first 7 days of culture in differentiation medium almost completely abrogated the maturation-related decrease of EpCAM (Fig. 1C). When the silencing of Vav1 was performed from the eighth to fourteenth days of differentiation, the effects on EpCAM were low even if the levels of this adhesion molecule was significantly higher than that found in progenitors grown for 14 days in PM (Fig. 1C). The analysis of cell morphology revealed that silencing of Vav1 during pancreatic differentiation prevented the appearance of aggregates of cells with a reduced size, in favor of cells with an elongated shape, similar to those of hBTSCs growth in control medium (Fig. 1D). The immunocytochemical analysis of differentiated hBTSCs revealed, as expected, a strong expression of insulin that was almost completely inhibited by silencing of Vav1 during differentiation (Fig. 1 D).

**Fig. 2:**
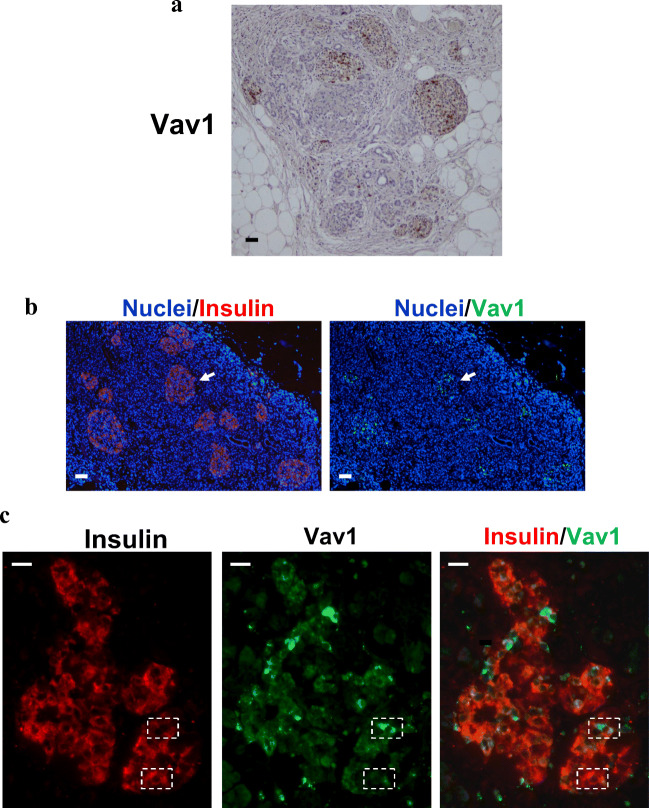
**In normal human pancreatic tissue Vav1 is present in insulin producing cells**. Representative images of formalin-fixed paraffin embedded normal pancreatic tissue sections subjected to immunohistochemical analysis with anti-Vav1 antibody (**A**) and to immunofluorescence using simultaneously antibodies against Vav1 (green staining) and insulin (red staining). Nuclei were counterstained with DAPI (**B**, **C**). In **B**, overlay of Vav1 (green) or insulin (red) with nuclei (blue) is reported. The arrow indicates a pancreatic islet positive for both Vav1 and insulin. In **C**, merged insulin/Vav1 staining is shown with co-localization resulting in yellow. The dashed rectangles identify β-cells with various levels of insulin and Vav1. Bar = 50 μm

In order to correlate Vav1 with the expression of insulin in mature β-cells, sections of human normal pancreas were subjected to histochemical analysis, confirming the absence of Vav1 in exocrine pancreas and revealing some Vav1 stained cells in Langerhans islets (Fig. 2A). Immunofluorescent histochemistry confirmed the presence of Vav1 in islets (Fig. 2B) and the Vav1/insulin co-staining revealed that insulin expressing cells contain variable levels of Vav1 (Fig. 2C).

### Vav1 Sustains the ATRA Induced Re/Trans-Differentiation of HPAF-2

**Fig. 3:**
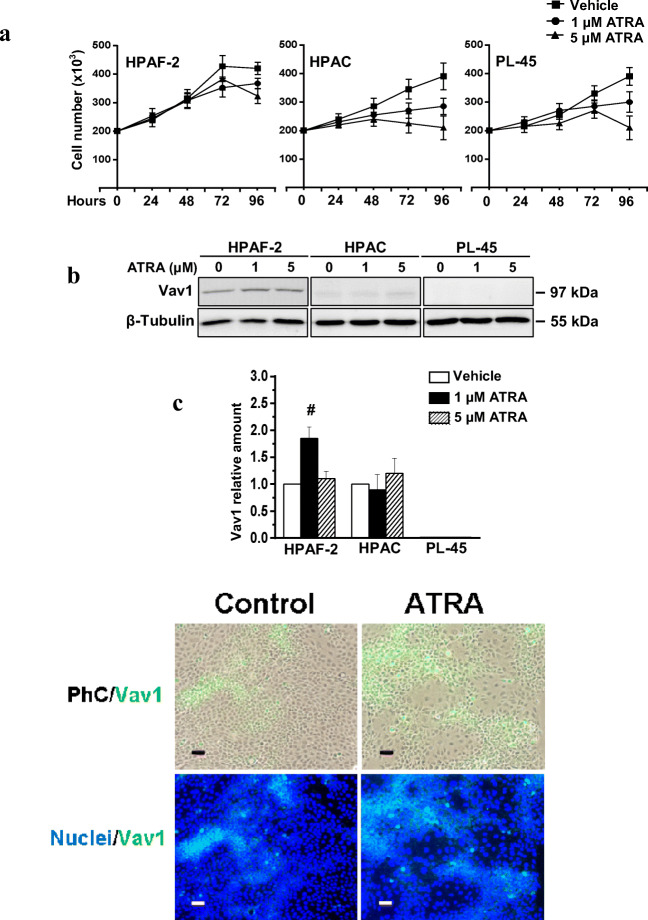
**ATRA up-modulates Vav1 in HPAF-2 cells.** In **(A**) the number of viable HPAF-2, HPAC and PL-45 pancreatic cancer cells cultured in the presence of different concentrations of ATRA for the indicated times. In (**B**) representative Western blot analysis with the reported antibodies of lysates from HPAF-2, HPAC and PL-45 cells grown for 96 h in the presence of ATRA at the indicated concentrations. In (**C**) levels of Vav1 as deduced from the densitometry of immunochemical bands normalized with β-Tubulin, used as internal control for equivalence of loaded proteins. Error bars indicate ± SD from a triplicate experiment. **#** *P <* 0.05*.* In (**D**) representative microscopy images of HPAF-2 cells grown for 96 h on glass dishes in control conditions or in the presence of 1 μM ATRA, subjected to immunocytochemical analysis with the anti-Vav1 antibody (green staining) and counterstained with DAPI (blue staining). Merged images of Vav1 staining with phase-contrast (PhC/Vav1) or with nuclear staining (Nuclei/Vav1) are shown. Bar: 50 μm

As both progenitor and mature neoplastic pancreatic cells are sensitive to ATRA administration [11, 33], we evaluated the involvement of Vav1 in the retinoid-induced differentiation and/or trans-differentiation of cell lines derived from a well differentiated (HPAC) or a poorly (PL45) differentiated primary PDCA, and from the metastatic site of a moderately differentiated pancreatic adenocarcinoma (HPAF-2), expressing different levels of Vav1. As reported in Fig. 3A, ATRA substantially reduced the number of viable HPAC and PL-45 cells in a dose dependent manner withouth affecting their low basal levels of Vav1 (Fig. 3B, C). At variance, ATRA only slightly affected the growth of HPAF-2 cells (Fig. 3A) but induced the expression of Vav1 (Fig. 3B, C). In particular, the increase of Vav1 was maximum with 1 μM ATRA, known to induce re-differentiation [11] or trans-differentiation [12] of pancreatic cancer cells.

**Fig. 4:**
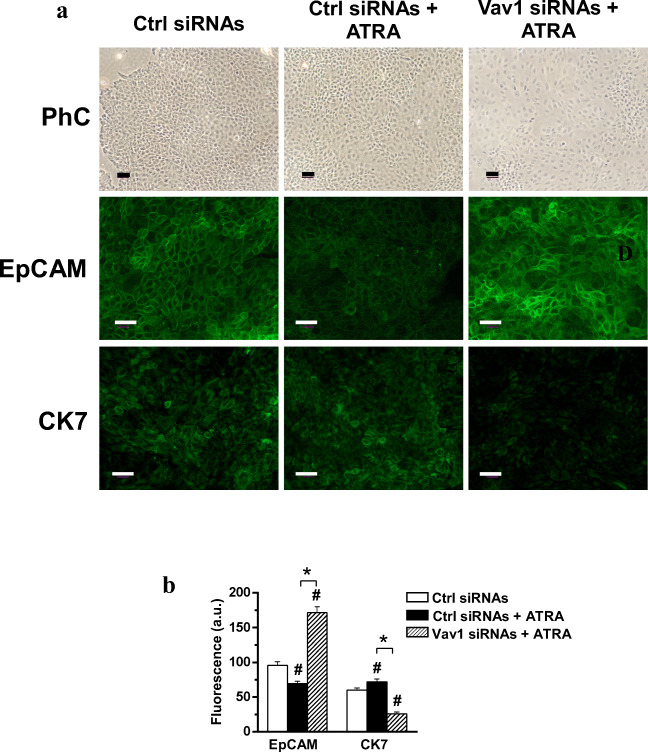
**Vav1 sustains the ATRA induced re-differentiation of HPAF-2 cells.** (**A**) Representative fluorescence microscopy images of HPAF-2 cells transfected with scramble siRNAs (Ctrl siRNAs) or with siRNAs specific for Vav1 (Vav1 siRNAs), grown for 96 h in the presence of 1 μM ATRA and subjected to immunocytochemical analysis with anti-EpCAM and anti-CK7 antibodies. Bar: 50 μm. (**B**) Fluorescence intensity of digitized images calculated by the ImageJ software. All the data are the mean of three separate experiments performed in triplicate ±SD. **#** *P* < 0.05 versus control; * *P* < 0.05 between bars

Since the HPAF-2 cell line [34] is a poorly differentiated spontaneous variant of HPAF-I that have a multipotent stem cell potential [11], the functional role of Vav1 up-modulated by ATRA was furtherly investigated in this in vitro system. The phenotype of HPAF-2 was firstly examined revealing retinoid-induced changes in both cell morphology and organization, suggestive of epithelial re-differentiation. In particular, ATRA induced in HPAF-2 the appearance of areas of clustered cells with a reduced size and showing high levels of Vav1, separated by regions with enlarged cells showing a lower Vav1 staining (Fig. 3D). The silencing of Vav1 during ATRA treatment strongly reduced the dimension of clusters in favor of the areas with elongated cells (Fig. 4A). As deduced by immunocytochemical analysis, the ATRA-induced decrease of the adhesion molecule EpCAM was counteracted by down-modulation of Vav1 (Fig. 4A, B). Furthermore, the ATRA induced expression of the epithelial marker cytokeratin 7 (CK7) was completely prevented by the silencing of Vav1 during agonist administration (Fig. 4A, B).

**Fig. 5:**
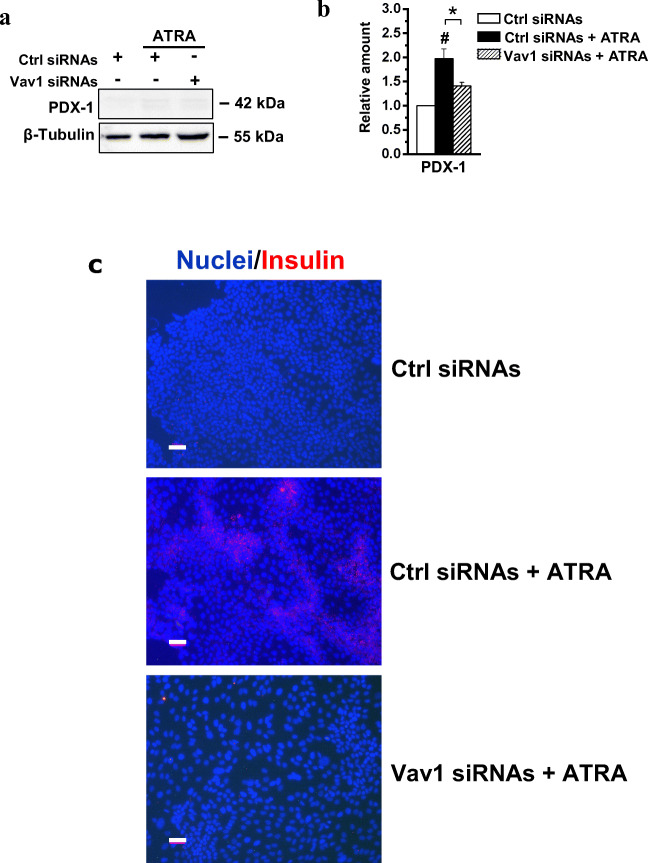
**Vav1 sustains the ATRA-induced trans-differentiation of HPAF-2 cells. (A)** Representative immunochemical analysis performed with the indicated antibodies of lysates from HPAF-2 cells transfected with siRNAs specific for Vav1 (Vav1 siRNAs) and cultured for 96 h in the presence of 1 μM ATRA. Scramble siRNAs (Ctrl siRNAs) was used as control. (**B**) Levels of PDX-1 as deduced from the densitometry of immunochemical bands normalized with β-Tubulin, used as internal control for equivalence of loaded proteins. All the data are the mean of three separate experiments performed in triplicate ±SD. **#** *P* < 0.05 versus control; * *P* < 0.05 between bars. (**C**) Representative immunofluorescence staining of HPAF-2 cells grown on glass dishes for 96 h under the same experimental conditions and subjected to immunocytochemical analysis with the anti-insulin antibody (red staining). Nuclei were counterstained with DAPI (blue staining). In all the examined experimental conditions, merged images of insulin expression with nuclear staining (Nuclei/Insulin) are shown. Bar = 50 μm

As HPAF-I was described to Trans-differentiate to functional β-cells after ATRA treatment [13], the effects of the retinoid on markers of pancreatic islets were evaluated in the HPAF-2 cell line. We assessed that the levels of the Pancreatic Duodenal Homeobox-1 (PDX-1), a transcription factor required for β-cells differentiation and maintenance of mature β-cells functions [35], is poorly expressed in HPAF-2 grown in control conditions and is slightly up-modulated by treatment with ATRA (Fig. 5A, B). The immunocytochemical evaluation of insulin revealed the ability of ATRA to induce in the HPAF-2 culture the appearance of areas with positive cells (Fig. 5C). Remarkably, both the ATRA induced increase of PDX-1 and insulin staining were counteracted by silencing of Vav1 during ATRA treatment (Fig. 5A, B, C).

**Fig. 6:**
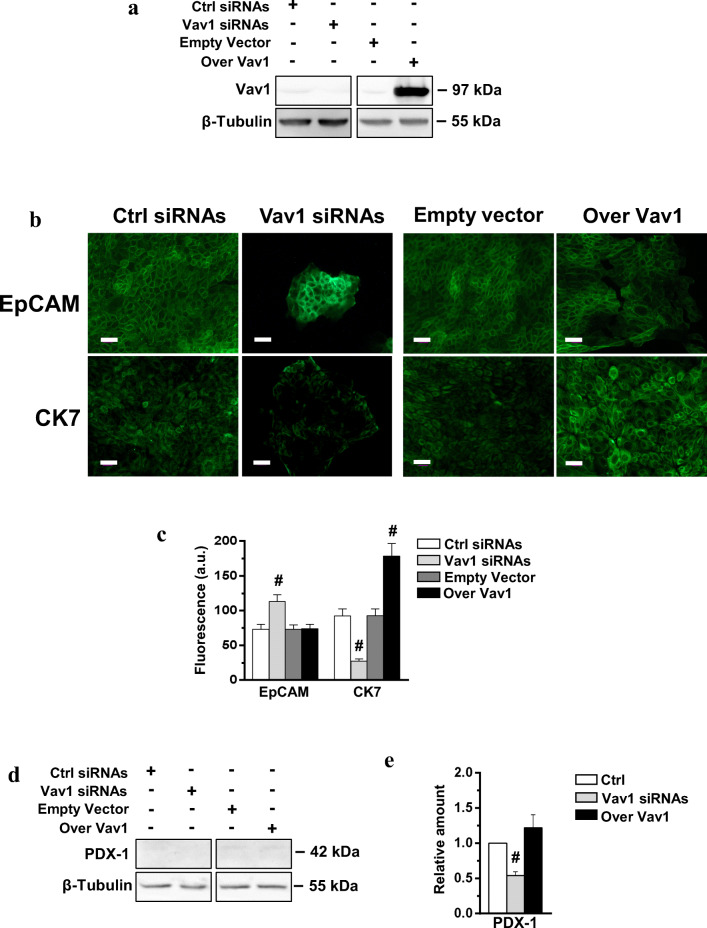
**Vav1 modulates re/trans-differentiation markers in HPAF-2 cells.** (**A**) Representative Western blot analysis with the indicated antibodies of HPAF-2 cells transfected with siRNAs specific for Vav1 (Vav1 siRNAs) or with a construct expressing the full-length human Vav1 (Over Vav1). Scramble siRNAs (Ctrl siRNAs) and an empty vector were used as controls. β-Tubulin was used as internal control for equivalence of loaded proteins. (**B**) Representative immunofluorescence staining of with the indicated antibodies of HPAF-2 cells in which Vav1 was either silenced or over-expressed. Bar = 50 μm. (**C**) Fluorescence intensity of digitized images calculated by the ImageJ software. (**D**) Representative immunochemical analysis performed with the indicated antibodies of lysates from HPAF-2 under the same experimental conditions. (**E**) Levels of PDX-1 as deduced from the densitometry of immunochemical bands normalized with β-Tubulin, used as internal control for equivalence of loaded proteins. All the data are the mean of 3 separate experiments ± SD. # statistically significant differences compared to transfection with respective controls (Ctrl)

To establish if the up-modulation of Vav1 in HPAF-2 cells can mimic the effects of ATRA, re/trans-differentiation markers were evaluated in HPAF-2 transiently transfected with a construct expressing the human protein (Fig. 6A). The over-expression of Vav1 induced the epithelial marker CK7, mimicking the effect of ATRA, while no effects on EpCAM was observed. On the other hand, decreased CK7 and increased EpCAM were revealed after silencing of Vav1 (Fig. 6B, C). Finally, we found that over-expression of Vav1 in HPAF-2 was ineffective on the levels of PDX-1 (Fig. 6D, E) and failed to induce insulin producing cells (data not shown), even if the disappearance of the low basal level of the transcription factor was shown when Vav1 was silenced (Fig. 6D, E).

## Discussion

The main result of this paper is the demonstration of a functional role of Vav1 in sustaining the pancreatic islet differentiation in vitro.

At variance with hematopoietic cells, in which Vav1 plays a well-known role in maturation and function [15–19], nothing is still known about its possible involvement in differentiation of solid tissues, in which its presence has been mainly correlated with the appearance of a tumor phenotype [20–25, 27]. It is well known that, in cells from APL, Vav1 is up-modulated by ATRA and is a crucial molecule in retinoid induced differentiation, participating in both cytoskeleton reorganization and protein expression [17–19]. ATRA is physiologically involved in differentiation of a number of epithelial tissues [2] and constitutes an attractive molecule for treatment of solid tumors, due to its ability to reduce growth and to induce a partial re-differentiation of tumor cells [11, 33, 36].

ATRA is required for both the development of pancreas and to maintain β-cell mass within islets, to prevent β-cell apoptosis, and to maintain proper insulin secretion from pancreatic β-cells [37, 38]. Furthermore, in cells from pancreatic cancer, one of the most aggressive tumor for which targeted therapies are not available [39], ATRA may act both as an anti-stromal agent [40] or as a direct anti-tumor molecule reducing cell growth, colony formation, and migration [41]. Finally, in vitro studies demonstrate that low doses of ATRA induce re-differentiation [11] and endocrine trans-differentiation of the PDAC derived HPAF cells, that have a multipotent stem cell potential, giving rise to functional insulin secreting cells [13].

Based on recent evidence that in adult human pancreatic duct glands (PDGs), distinct niches of progenitor cells constitute a natural source of β cells for the purposes of regenerative medicine [42–44] and that PDGs might have a central role in the development of PDAC [45], human biliary tree stem/progenitor cells and PDAC-derived cell lines were used to assess whether ATRA induces the differentiation of both cell systems towards a pancreatic islet fate by up-modulating the expression of Vav1.

We firstly evaluated the contribution of Vav1 to ATRA-induced maturation to β-cells of normal precursors. We used the well-established model of pancreatic differentiation constituted by hBTSCs cultured with a specific differentiation medium containing ATRA [30]. We demonstrated that these precursor cells express Vav1 that was up-modulated during their pancreatic differentiation and resulted essential for the differentiation-related decrease of the stem cell marker EpCAM [30]. The evaluation of cell morphology and of insulin expression established that adequate levels of Vav1 are necessary for functional maturation of hBTSCs towards insulin-producing β-cells. The time-dependent decrease of Vav1 expression observed during differentiation of the multipotent hBTSCs to hepatocytes and cholangiocytes [30] highlighted the specific involvement of Vav1 in differentiation of these precursors to pancreatic β-cells, possibly as part of the intracellular signaling activated by ATRA.

Once established that Vav1 is involved in vitro in maturation of bilio-pancreatic precursors to insulin producing cells, we tried to assess if the Vav1/insulin relationship also exists in the adult pancreas. We first revealed the presence of Vav1 expressing cells in the endocrine pancreas and, in particular, we found that insulin-producing cells express variable Vav1 levels. As β-cells are heterogeneous in their maturation levels, insulin secretion and specific phenotype [46, 47], the presence of high levels of Vav1 in cells expressing low insulin and the low Vav1 staining in cells showing high insulin content suggest that Vav1 can be involved in the earlier stages of the maturation process of insulin producing-cells and/or that different levels of Vav1 identify different metabolic/subtypes of β-cells.

Moreover, we found that ATRA induced a significant increase of Vav1 in the HPAF-2 cell line, a spontaneous variant of HPAF-I with a stem cell potential, able to undergo re-differentiation [11] or trans-differentiation to insulin producing cells [13] in response to the retinoid. In this cell model, we found that Vav1 is essential for the ATRA induced decrease of the adhesion molecule EpCAM, known to be a marker of pancreatic adenocarcinoma stem cells [48]. This suggests that, at least in HPAF-2 cells, the expression of Vav1 counteracts that of molecules with a well-known role in the malignancy of solid tumors [49, 50]. In HPAF-2, as observed in HPAF-I [11] and in other tumor cells [33, 36], ATRA induced a partial epithelial re-differentiation, that we have found almost entirely determined by the presence of adequate levels of Vav1. Also considering that the over-expression of Vav1 is sufficient to up-modulate the epithelial marker CK7, our data suggest that the role of Vav1 in pancreatic tumor cells could be reconsidered on the basis of its ability to act as a direct promoter of an epithelial-like phenotype.

To assess if ATRA induces endocrine trans-differentiation of HPAF-2 cells, markers of functional β-cells were explored, revealing that ATRA induced the expression of PDX-1, a transcription factor crucial for differentiation of and function of β-cells [35, 51, 52] and able to promote the maturation of hBTSCs to insulin producing cells [53]. The increase of PDX-1 was accompanied by the appearance of massed cell expressing insulin, and both these events were completely dependent on the presence of proper Vav1 levels during ATRA treatment.

Overall, our data demonstrate that the in vitro achievement of insulin-producing cells as a result of ATRA-induced differentiation of precursors or pancreatic cancer cells is almost completely dependent from Vav1, whose expression is in turn regulated by ATRA. As the forced expression of Vav1 was unable to mimic the effects of ATRA on both PDX-1 and insulin, but is essential for the ATRA induced increase of both molecules, we can deduce that Vav1 supports the transcriptional machinery activated by the retinoid, as demonstrated in APL cells [17–19]. Finally, we can conclude that Vav1 is unable to induce the maturation of β-cells but is an essential molecule in the differentiation process of both normal and tumor precursors activated by ATRA.

The relationship between retinoids deficiency and the appearance of Type 1 diabetes mellitus, caused by reduction of the β-cell mass and function [54] as well as the evidence of ATRA-induced restoration of Langerhans islet into diabetic rats [55] substantiate the crucial role of retinoids in β-cell formation in vivo. It is intriguing that in recent years the therapeutic approach to diabetes mellitus have explored new tools for the restoration of fully functional β-cells based on a set of strategies which include differentiation of multipotent stem cells [56–58], expansion of existing pools of cells, either from donors or by differentiating stem cells, and trans-differentiation of exocrine and endocrine pancreatic cells other than β-cells [59]. In this context, efforts directed to identify molecules that can trigger β-cell differentiation and replication look with renewed interest at the ATRA mediated signaling.

Taken together, the results of this study shed light on the molecules involved in the differentiation mechanisms activated by ATRA in pancreatic tumor cells or normal precursors to induce functional β-cells. Both mechanisms emphasize the essential role of Vav1, and suggest that this molecule can be a sensitive marker useful for monitoring the use of ATRA in new therapeutic strategies for diabetic patients.
